# Diagnostic options for coronavirus disease 2019 (COVID-19)

**DOI:** 10.1017/ice.2020.168

**Published:** 2020-04-23

**Authors:** Yuanyuan Xiao, Zhong Peng, Caixia Tan, Xiujuan Meng, Xun Huang, Anhua Wu, Chunhui Li

**Affiliations:** 1 Xiangya Hospital Central South University, Changsha, Hunan Province, China; 2 State Key Laboratory of Agricultural Microbiology, College of Veterinary Medicine, Huazhong Agricultural University, Wuhan 430070, Hubei, China; 3 National Clinical Research Center for Geriatric Disorders (Xiangya Hospital), Changsha, Hunan Province, China

*To the Editor*—The COVID-19 pandemic is posing a great challenge to global health and economy. Early accurate diagnosis plays a key role in fighting the disease. However, the diagnosis might be missed because of false-negative tests due to the insufficient sensitivity of the only test that detects SARS-CoV-2 or coinfection with other viruses. These false-negative results will affect clinical management decisions as well as control of the epidemic. Therefore, broader viral tests should be given to patients suspected to have COVID-19. Here, we discuss the existing methods of diagnosing COVID-19.

## Nucleic acid amplification tests (NAAT)

The current first choice for the etiological diagnosis of COVID-19 is based on detection of unique sequences of virus RNA by real-time reverse-transcription polymerase chain reaction (rRT-PCR).^1^ The PCR test is appropriate for the acute phase of illness; however, cases of missed diagnoses have already been reported using this method.^2,3^ Recently, related research shows that the COVID-19-RdRp/Hel rRT-PCR test is highly sensitive and specific, which might help to reduce the false-negative rate and would be significantly useful for detecting specimens with low viral loads.^3^ Thus, in terms of technical and financial support, the current rRT-PCR testing available is relatively optimal for SARS-CoV-2 screening of suspected cases.

## Viral sequencing

The application of next-generation sequencing may be an accurate diagnosis method for SARS-CoV-2, including metagenomics, hybrid capture-based sequencing, and amplicon-based next-generation sequencing.^1,4,5^ These 3 approaches show a higher sensitivity than conventional RT-PCR, and they can meet the need for secondary detection, diagnosis confirmation, and large-scale detection of RT-PCR false-negative results.^5^ However, high cost is currently an important obstacle to more widespread use of virus sequencing.

## Serological testing

For patients with COVID-19, detectable SARS-CoV-2 antibodies are mainly divided into IgM and IgG. In general, most of SARS-CoV-2–specific IgM antibodies can be detected 3–5 days after onset, and during the recovery period, IgG antibody titers are ≥4 times higher than in the acute phase.^4,6^ An antibody test is appropriate for the convalescence phase of COVID-19 in case of a symptomatic infection. This method, however, is susceptible to the presence of some interfering substances in the blood sample (eg, rheumatoid factor, nonspecific IgM, etc), and therefore, it has a very high false-positive rate. Hence, SARS-CoV-2–specific IgM or IgG antibody testing can be used as a diagnostic standard for COVID-19 in the case of a negative NAAT, when 2 dynamic tests are required.^1,6^

## Rapid antigen tests

In theory, rapid antigen tests have the advantages of fast detection speed and low cost, but as yet they have poor sensitivity and specificity for detecting coronaviruses (except MERS).^7^ Moreover, it is almost impossible to identify patients in the incubation period of infection, which is to say that antigen tests cannot be used as the sole basis for the diagnosis or exclusion of COVID-19. A pre–peer-reviewed article reported that a fluorescence immunochromatographic assay is an accurate, rapid, early and simple method for detecting the nucleocapsid protein of SARS-CoV-2 in nasopharyngeal swab samples and urine samples for the diagnosis of COVID-19.^8^ This claim requires further investigation.

## Imaging examinations

Because lung abnormalities may appear ahead of clinical manifestations and positive NAAT, some studies have recommended that early chest computerized tomography (CT) be used to screen suspected cases of COVID-19.^2,4,9,10^ Furthermore, pneumonia manifests with chest CT imaging and suggests the evolution and prognosis of COVID-19.^2,10^ Nevertheless, due to the highly contagious nature of SARS-CoV-2 and the risk of transporting critically ill patients, the choice to conduct a chest CT scan in patients with suspected or established COVID-19 is made infrequently. In addition, lung ultrasonography may have great utility in managing COVID-19 pneumonia due to its safety, repeatability, absence of radiation, low cost, and point-of-care use.^9^ For cases in which pulmonary ultrasound is not sufficient to answer clinical questions, a chest CT is needed.

In summary, combining assessment of imaging features with clinical and laboratory findings could facilitate early diagnosis of COVID-19. Here, we have systematically summarized the various diagnostic methods for SARS-CoV-2. More importantly, this work offers practical options for diagnosing COVID-19. Our experience may help clinicians make better decisions in the effort to become victorious over SARS-CoV-2.
